# Highlighting the impact of social relationships on the propagation of respiratory viruses using percolation theory

**DOI:** 10.1038/s41598-021-03812-9

**Published:** 2021-12-21

**Authors:** Jean-François Mathiot, Laurent Gerbaud, Vincent Breton

**Affiliations:** 1 Laboratoire de Physique de Clermont, CNRS/IN2P3, Université Clermont Auvergne, 63000 Clermont-Ferrand, France; 2 Institut Pascal, CHU Clermont-Ferrand, SIGMA Clermont, CNRS, Université Clermont Auvergne, 63000 Clermont-Ferrand, France

**Keywords:** Respiratory tract diseases, Lifestyle modification, Epidemiology

## Abstract

We develop a site-bond percolation model, called *PERCOVID*, in order to describe the time evolution of all epidemics propagating through respiratory tract or by skin contacts in human populations. This model is based on a network of social relationships representing interconnected households experiencing governmental non-pharmaceutical interventions. As a very first testing ground, we apply our model to the understanding of the dynamics of the COVID-19 pandemic in France from December 2019 up to December 2021. Our model shows the impact of lockdowns and curfews, as well as the influence of the progressive vaccination campaign in order to keep *COVID-19* pandemic under the percolation threshold. We illustrate the role played by social interactions by comparing two typical scenarios with low or high strengths of social relationships as compared to France during the first wave in March 2020. We investigate finally the role played by the α and δ variants in the evolution of the epidemic in France till autumn 2021, paying particular attention to the essential role played by the vaccination. Our model predicts that the rise of the epidemic observed in July and August 2021 would not result in a new major epidemic wave in France.

## Introduction

Before the development of an approved vaccine for the prevention of *SARS-CoV-2* infection, and in the absence of curative pharmaceutical options for the treatment of the *COVID-19* pandemic, *Non Pharmaceutical Interventions (NPIs)* have been the only available policy levels to reduce the virus transmission^1^. In this respect, mathematical modelling has proven to be an important tool in order to guide policy makers for taking the appropriate decisions regarding these *NPIs*. Reciprocally, the wealth of epidemiological data made publicly available since the beginning of the pandemic provides a unique opportunity to understand the main characteristics of the existing models on a deeper ground.

The modelisation of epidemics propagating through respiratory tract or by skin contacts in human populations relies on two observations: they propagate through the formation of clusters of increasing size and this propagation is mainly governed by the density and the intensity of the social relationships in the population under study. In the case of the *COVID-19* pandemic, there is a slow continuous transmission combining day-to-day interactions, with highest proportion of asymptomatic cases, and accelerations characterized by the links between super spreader events, combining a larger number of human interactions with high viral emission^2–4^. Although this has been widely recognized during the *COVID-19* pandemic, these two observations are however not properly taken care of in most of *COVID* epidemic simulations.

The *SEIR* compartmental type models^5^ rely on a global approach with no information whatsoever on the formation of clusters. They only rely on the total density of susceptible, exposed, infected or recovered persons over the whole territory under study, independently of their social surrounding. The role of social relationships is embedded in a very general parameter—the so-called reproduction number *R*_0_—which incorporates also epidemiological information like the intrinsic infectiosity and infectious period duration. The characteristic length scale of these models is thus not small enough to be able to follow the epidemic on the long term when the local environment of each person starts to be most important and governs the virus propagation^6–8^.

The *Multi-Agent System* models are very detailed simulations of a restricted territory—such as a town—with a rather large granularity—like a building—and a small time scale—like an hour. They require evaluating multiple scenarii and therefore large computing capacities^9^. It is therefore very difficult to extract any generic behavior at the level of a large territory and to identify collective effects like abrupt transitions. The characteristic length scale of such models is thus much too small to follow the epidemic over a long period of time.

These remarks motivated our choice to consider a realistic model at a mesoscopic scale, based on percolation theory^10^. The formation of clusters is the way percolation models operate, while collective behaviors lead to a sharp transition, the so-called percolation transition. This transition occurs from a *non-percolation* regime with many small clusters to a *percolation* regime with a large cluster at a macroscopic scale, *i.e*. with a typical size of 50% of the population or larger. Compared to previous attempts at modeling the *COVID-19* pandemic using percolation-type models^11^, we advocate in this study their formulation on a *network of social relationships*, emphasizing the role of these relationships in the propagation of the epidemics rather than the geographical proximity of persons. The interpretation of percolation theory in terms of social relationships was already applied to other contexts^12^. The characteristic mesoscopic scale of such model enables also to follow the epidemic over a very long period of time and the *COVID-19* pandemic provides a tremendous testing ground of its validity for about two years.

We describe in this study the general properties of the *PERCOVID* percolation model, detail its construction and show how the virus propagation displays a sharp transition between a percolation and non-percolation regime depending on the density and intensity of social relationships. The analysis, within our model, of the *COVID-19* pandemic in metropolitan France is presented: the raise of the first wave in March 2020, the impact of the first lockdown, the second wave during the fall 2020 followed by the second lockdown, the third wave in spring 2021, as well as the influence of the curfews and of *Super-Spreading Events* (*SSE*) in this evolution. We illustrate the role of social relationships by comparing the dynamics of the epidemic propagation for two configurations with low and high strengths of social relationships as compared to France, for the beginning of the epidemic. We also detail the role played by a progressive vaccination campaign in 2021 together with the influence of the propagation of the α and δ variants. All necessary additional details on the *PERCOVID* model are given in Supplementary Information.

## Methods

We constructed *PERCOVID* following the main characteristics of an epidemic propagating through respiratory tract or by skin contact in human populations. It is defined on a lattice in *D* dimensions with, as usual in site-bond percolation theory, sites connected by links. The only geographical proximity which is relevant to the propagation of the virus corresponds to persons sharing the same household. Each site of the network is therefore identified with one, two, or three and more persons belonging to the same household, with a distribution according to the territory under study. The connexions between the sites are represented by the bonds between them. They correspond to the daily social interactions between any person in one household with any person in the neighboring household on the lattice.

We need to emphasize here that a neighbor on our (social) lattice is not necessarily a geographical neighbor: indeed, two persons can have close social interactions while living in geographically distant locations. These social relationships do control the propagation of the epidemic and are embedded, by construction, in *PERCOVID*. They correspond for example to interactions in the professional and school circles, during extraprofessional activities with friends or with the family living outside the household. All these activities should not be considered however on the same footing, as far as the propagation of the epidemic is concerned. We therefore consider a first circle of so-called *essential* activities corresponding to the closest neighbors on the lattice, while *less-essential* activities are associated to a second circle represented by next-to-closest neighbors.

The richness of these daily social relationships depends on the dimension *D* of the lattice. For *D* = *1* for instance, this corresponds to very limited relationships with only two neighbors. For *D* = *2*, we start having a second circle of less essential relationships, with four next-to-closest neighbors, and four closest neighbors for the first circle. In order to account for the observed social behaviors in different European countries, we consider in this study a three-dimensional lattice. This implies a maximum of six possible daily interactions in the first circle which may lead to a transmission of a given respiratory virus, and twelve more for the second circle. The situation with *D* = *4* may correspond to exceptional situations like *Super Spreading Events* for instance, with very rich social relationships within a specific group of persons over a limited period of time.

Each site of the lattice is occupied with a probability *p*. Since the links over the lattice are associated to daily social relationships, this probability is therefore also identified with the mean density of these relationships on the territory under study, normalized to one for a maximum of *18* interactions in both the first and second circles. The value for *p* is adjusted in our model to reproduce the average number of daily social relationships in France and various European countries^13,14^. Following the classification already used in the *SEIR* epidemic models, any person belonging to a household can be either Susceptible, Exposed, Infected or Recovered. Once infected, a person is either pre-symptomatic, symptomatic or asymptomatic. In the case of *SARS-CoV2*, it can also be vaccinated since January 2021 in France. The model can also take into account the concurrent propagation in the population of up to nine variants.

The time evolution of the epidemic is governed by the probability for an infected person to infect a susceptible one in a daily social interaction. This interaction can mainly occur either with one member of the same household or with someone in its first or second circle of neighbors. This probability depends on two very different parameters: the first one, of epidemiological nature, is associated to the intrinsic properties of the virus and to the viral load of the infected person. It corresponds to the ability of the virus to infect one susceptible person during a social contact, independently of any other considerations. We call it *infectiousness* and denote it by *r*, with values ranging from 0 to 1. The second parameter, of social nature, is related to the *intensity* of the social relationships in the territory under study and is denoted by *q*. It is independent of the *density* of these social interactions represented by *p*. It is related, on the one hand, to the social behavior of the population and, on the other hand, to the social distancing measures taken by the authorities and adopted by the population for reducing the propagation of the virus. A so-called temperature effect is introduced to reduce both the infectiousness of the virus and the intensity of social interactions during summer as life style evolves to more outdoor activities less favorable to virus transmission^15^. In order to account for exceptional interactions outside the first or second circle, the model also opens the possibility for any person to interact episodically with any person anywhere in the territory under study.

Once a susceptible person is infected with the probability *q.r*, an internal clock is started to follow the disease evolution. This evolution is governed by the infectious time *τ*_*i*_ when the infected person starts to be contagious, and the recovered time *τ*_*r*_ when it recovers (or dies). At each iteration step, the status of each member of each household is evolved according to the previous principles, as detailed in Supplementary Information.

## Results

### Phase diagram

**Figure 1:**
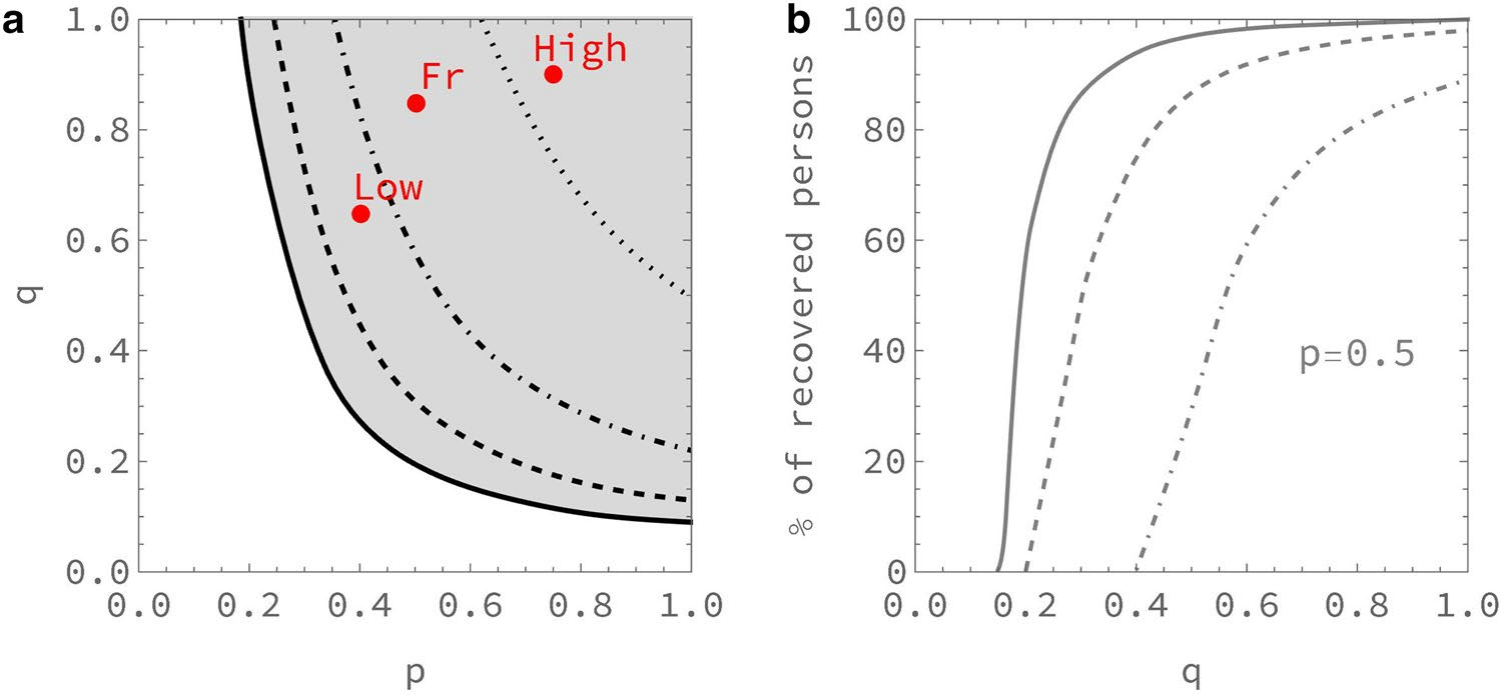
(**a**) Phase diagram of PERCOVID for the SARS-CoV-2 initial strain. The full line shows the limit between the non-percolation and percolation zones, in white and grey respectively in absence of vaccination while the dashed (25% coverage), dot-dashed (50% coverage) and dotted (75% coverage) curves show how the vaccination reduces the percolation zone. The points labeled “Fr”, “Low” and “High” correspond to the expected position associated to the social behavior in France, or to lower or higher strengths of social relationships respectively, at the beginning of the epidemic. (**b**) Percentage of recovered persons at the end of the epidemic, for the case of metropolitan France (*p* = 0.5), with three different vaccination rates (solid line 0%, dashed line 25% and dot-dashed line 50%) as a function of the intensity *q* of the social relationships.

The percolation transition for a given respiratory virus can be easily identified in a phase diagram in the *(p,q)* plane. We define this transition by the critical values of *p* and *q* for which the total number of recovered persons at the end of the epidemic exceeds 50% of the (non-vaccinated) population of the territory under study. Figure 1a displays the phase diagram corresponding to the *SARS-CoV2* initial strain, for four different vaccination rates. We indicate also on this figure the positions corresponding to populations with lower or higher strengths of social relationships as compared to France at the begining of the *COVID-19* pandemic.

According to our model, France and the two configurations labeled *Low* and *High* are all in the percolation zone when the *COVID-19* pandemic started, before any *NPI* is enforced. However, their position reflects the difference in the social behavior of the corresponding populations. As we shall see below, the impact of the first *COVID-19* pandemic wave is significantly different for these three configurations. The relative position of these configurations with respect to the critical line for a given vaccination rate and a given value of the intensity of social relationships enables us also to predict when the herd immunity should be reached in this country, *i.e.* in our study when the non-percolation zone should be reached. The corresponding phase diagram associated to the δ variant is indicated on Supplementary Figure S1. Note that the conditions to reach the herd immunity do not depend only on the epidemiological parameters like the infectiousness of the virus or the contamination duration of an infected person, but also on the social behavior of the population through the value of the density and intensity of their social relationships. As a consequence, the rate of vaccination needed to achieve herd immunity does depend explicitly on *q*.

Figure 1b illustrates the sharpness of the percolation transition, *i.e.* how varying *q* around its critical value, for a given value of *p*, can have a huge impact on the dynamics of the epidemic. While the density *p* of the social tissue is a cultural variable independent of any governmental intervention, wearing a mask and refraining from physical contact translates, in our model, into reducing *q*, the intensity of social interactions. This figure also shows how this transition depends on the vaccination rate. Note that for large values of this rate, the percentage of recovered persons when *q* tends to 1 is not 100% since there remains some islands of isolated sites on the lattice which cannot be reached by the virus.

### Time evolution of *COVID-19* in France

**Figure 2:**
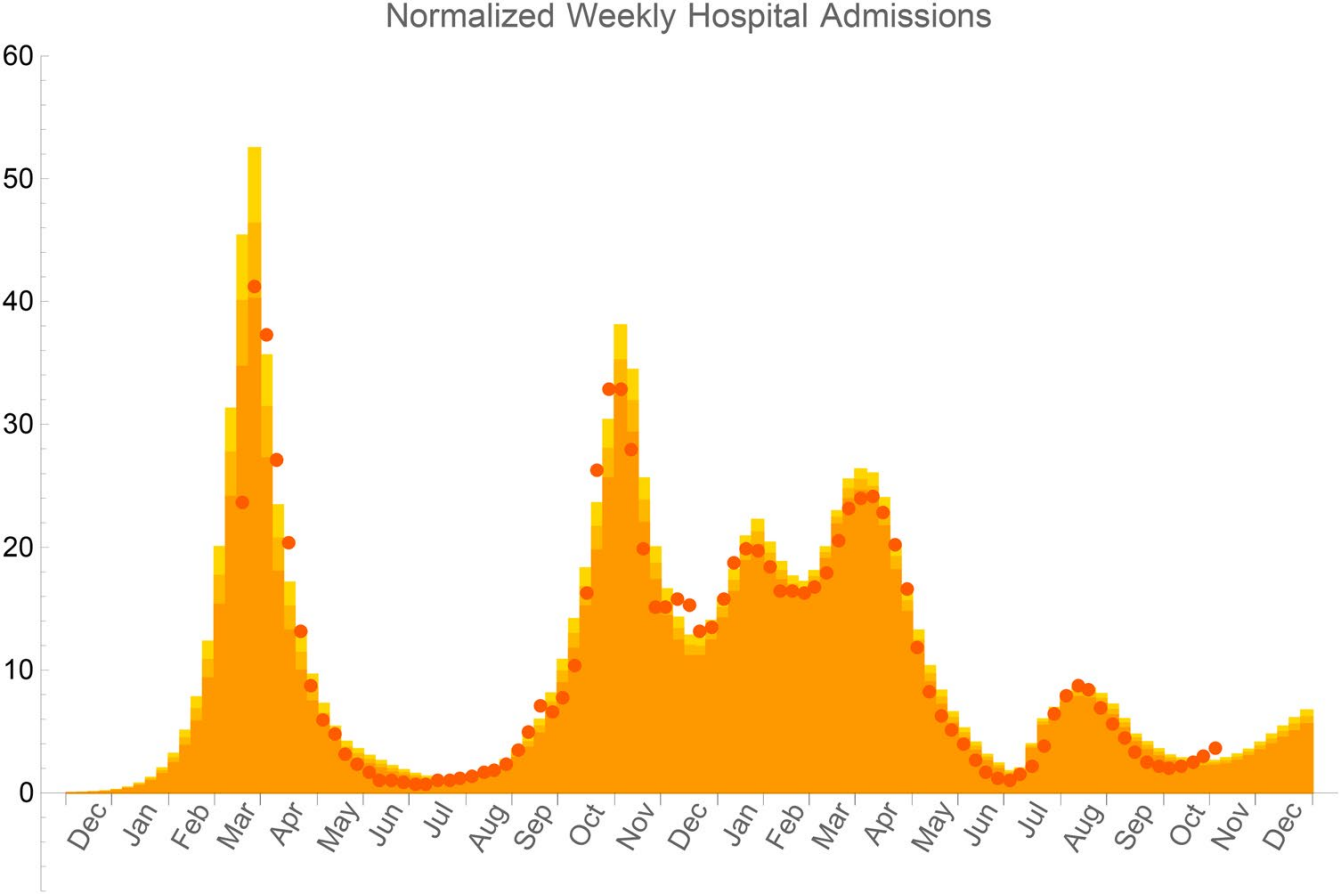
Number of weekly hospital admissions normalized to 50,000 households. Orange bars correspond to PERCOVID model prediction averaged over ten simulations and red points to the epidemiological data from Santé Publique France^18^. Statistical errors at 95% CL are represented by slightly different orange shades.

Since the possible saturation of hospital facilities is the main reason for deciding lockdowns, we choose as first epidemic indicator the number of weekly hospital admissions normalized to 50,000 households. It also better reflects the epidemic evolution than the number of cases that depends on the screening policy. Figure 2 shows how our model can account for the normalized weekly hospital admissions due to *COVID-19* pandemic in metropolitan France from December 2019 to October 2021. The simulation is done on a lattice representing the number of metropolitan French households at a scale of *1/10*. It takes into account the influence of the progressive vaccination campaign as well as the propagation of the α and δ variants. *NPIs* endhorsed by the French government are accounted for by changing *q* from its initial value together with possible restrictions to the access to the second circle and to the mobility and contacts ouside the first and second circle, while keeping *p* constant. All details on the choice of the parameters of the simulation are given in Supplementary Information.

Our model agrees with the early circulation of *SARS-CoV-2* in France as documented from population-based cohorts^16,17^. At the scale of metropolitan France, it corresponds to 140 (symptomatics and asymptomatics) infected persons on December 1, 2019. The model confirms the efficiency of the two national lockdowns from March *17*th to May *11*th*, 2020* and from October *30*th to December *15*th*, 2020* to slow down the virus propagation. The influence of the curfew starting at *6 pm* is also seen from the decrease of the hospital admissions in February 2021. The subsequent third wave in March/April 2021 is entirely due to the rapid propagation of the *α* variant, with a larger infectiousness in a context of reduced vaccination coverage.

**Figure 3:**
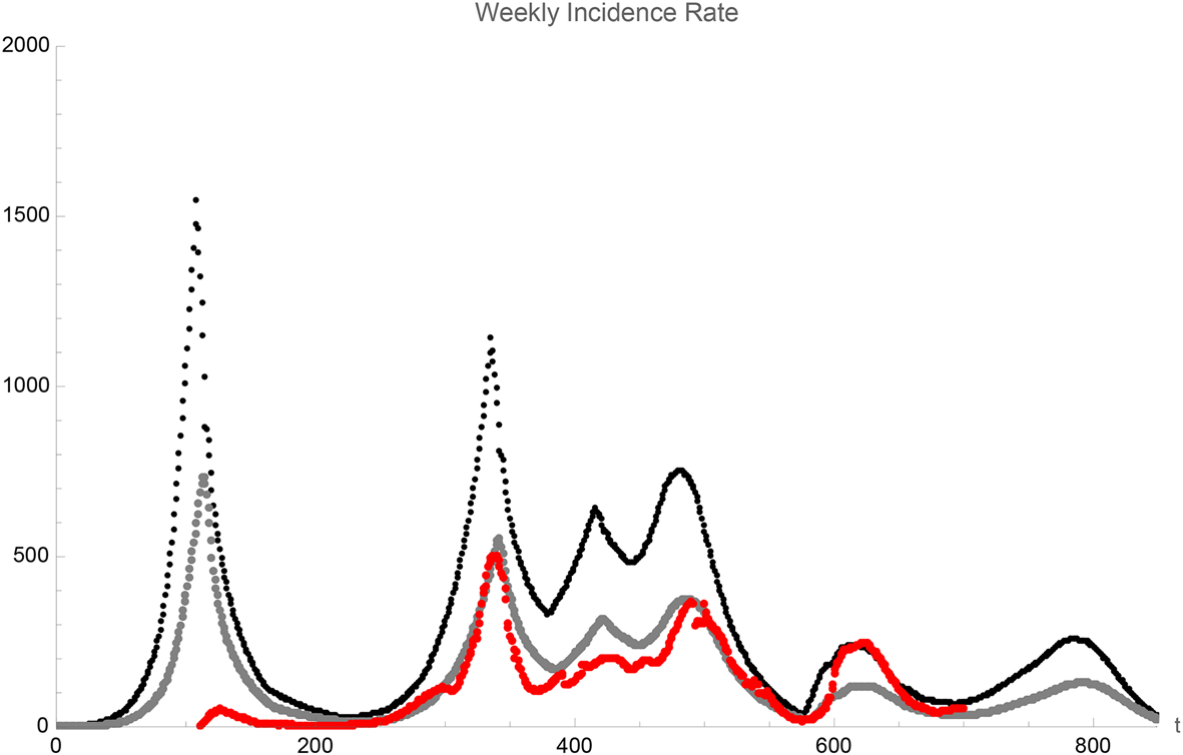
Incidence rate, sumed up over the preceeding week, as calculated in our model with symptomatics as well as asymptomatics infected persons (black points) or with symptomatic ones only (gray poins) as compared to data from^18^ (red points). The origin of time, t = 0, corresponds to December 1,2019.

**Figure 4:**
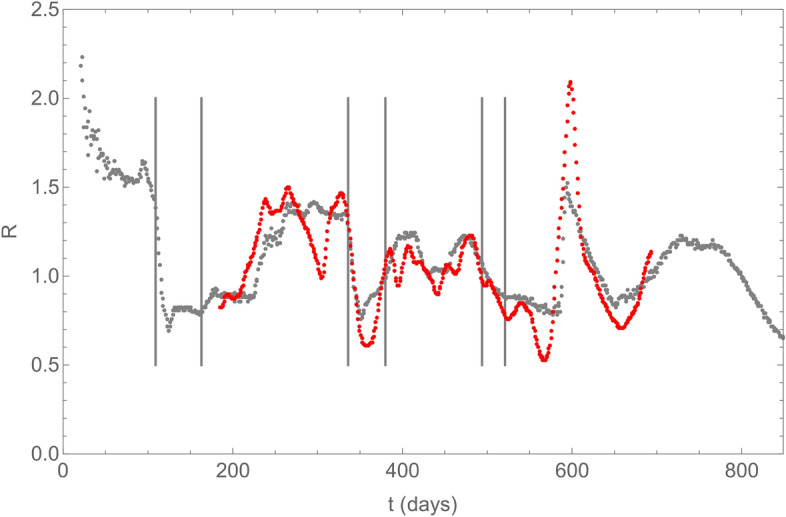
Time evolution of the effective reproduction number R during the COVID-19 pandemic in France. The origin of time, t = 0, corresponds to December 1,2019. The vertical bars correspond to the beginning and end of the three confinement periods in France. The black points correspond to the prediction of our model, while the red points correspond to the data from Ref.^18^.

Although the δ variant quickly represented about 100% of the infected persons due to its large infectiousness at the end of summer 2021, our model predicted only a moderate increase of the number of hospital admissions in metropolitan France in July and August 2021, before a rapid decrease in September 2021. This absence of a new wave in summer is entirely due to the fact that there are not enough susceptible individuals in the social local environment of any infected one. Due to the natural immunity already acquired by the recovered persons, and the growing vaccination coverage of the population, the virus could not therefore propagate anymore on a long length and time scale. This corresponds, in terms of the phase diagram discussed above (see Supplementary Figure S1), to the non-percolation regime. The slight increase of weekly hospital admission in october 2021 can only be accounted for in our model by a sizeable increase of the intensity of social relationships, as indicated in Supplementary Table S7. This is in qualitative agreement with the observed relaxation in social distancing measures in localized super-spreading events, in particular in view of the inhomogeneity of the vaccination coverage observed in France, ranging from 75 to 95% of the adult population^18^. As shown by the values of both the incidence rate and the effective reproduction number on Figs. 3 and 4 respectively, this increase will again not result in a major new wave in France.

Figure 3 displays the COVID-19 incident rate that was calculated in our model for all infected persons as well as only symptomatic ones and compares it to the official incident rate^18^ that was computed from the number of positive PCR tests collected at a national level in the last seven days. The generalized use of PCR tests targeting all citizens, whether or not showing symptoms of COVID-19 infection, started only in August 2021 and the enforcement of the sanitary pass by the French government. From June 16th 2020 (day 200 of the simulation) to July 21st 2021 (day 600), the number of weekly tests has grown from 230.000 to 2.600.000 for an overall population of 67 millions. Due to the delayed availability of PCR tests, the calculated incidence rate during the first wave in March 2020 is largely above the official incidence rate, in agreement with the very large impact of the first wave on the hospital admissions.

As PCR tests were more targeted to confirm COVID-19 infection during the second and third waves in November 2020 (around day 320) and April 2021 (around day 500), the prediction of our model for symptomatic persons only is in good agreement with the official data. From mid summer 2021 (around day 620) and the extensive testing of a significant fraction of the population, our model including both symptomatic and asymptomatic cases is in overall agreement with the official data. Note that the comparison between both observables on Figs. 2 and 3 for the epidemic wave observed in August 2021 enables to constrain the rate of infected persons requiring hospital admission for the δ variant. This rate is identical to the one estimated for the initial strain, as documented in Supplementary Table S1.

### The effective reproduction number R

Knowing for each person in each household the whole history of its infection pattern, thanks to the triggering of its internal clock when it gets infected, we can directly calculate the so-called effective reproduction number *R* at any time, as shown on Fig. 4. This number corresponds to the average over the number of secondary infections an infected person can induce during the period it is contaminant. The basic reproduction number *R*_0_, corresponding to the value of *R* at the begining of the epidemic, is not a free parameter in our model, as it is the case in the commonly used *SEIR* models. It depends implicitly on many parameters of quite different nature, like *D, p, q, r* and *τ*_*r*_-*τ*_*i*_.

This figure shows expected features like the rapid decrease of *R* during the beginning of the first and second confinement periods. Note also the peak in *R* just before the first confinement period. It corresponds to the extra infections due to the *SSE* in Mulhouse^17^. Since the value of *R* was already high at that time, the *SSE* had no large influence on the evolution of the epidemic at country scale. This figure shows also two interesting features as far as the propagation of the variants is concerned. Firstly, the third confinement period had a limited impact for reducing R. It also shows that this observed reduction already started *before* the occurrence of this third confinement period, indicating that the propagation of the α variant was already decreasing in metropolitan France at that time. Secondly, the rapid growth of *R* around day 600 illustrates the very rapid growth of *SARS-CoV-2* infections due to the highly contagious δ variant. However, our model shows that the progress in the vaccination coverage of the french population, together with the acquired natural immunity will limit very efficiently its impact, leading to a rapid decrease of *R*. All these features are nicely confirmed by the data from the french authorities^18^, apart from large dips in the data which do not correspond to any restrictions in the social behavior of the population and may just be due to a change in the way data from infected persons have been collected. The value of R in december 2021 (around day 750) is compatible with a slight increase of infections, as confirmed by the observed increase of weekly admission rate indicated on Fig. 2. The extension of our simulation till the end of winter (around day 850) shows however that the effective reproduction number will first stay slightly above 1 before decreasing rapidly again.

We indicate on Supplementary Figure S2 the number of secondary infections one infected person has induced. It shows that although about 45% of infected persons have not infected anyone, the number of secondary infections one infected person can induce can be as large as 5 or 6. This indicates that when the virus still propagates on a large space scale, even at a low level as it is the case in France in Autumn 2021, any local super-spreading event associated to a high value of the intensity of social relationships in a population with a locally reduced vaccination coverage can easily induce a sizeable increase of infections as it is observed on Fig. 2 and confirmed by the raise of the effective reproduction number on Fig. 3. This local increase of infections will not have however an important impact on a large time scale provided the overall vaccination rate of the population, with a third dose of vaccin if necessary, is large enough to stay outside the percolation zone.

### The role of variants in SARS-CoV2 epidemic evolution

Having adjusted our model parameters to reproduce *COVID-19* pandemic time evolution in metropolitan France, we can investigate the role of the various variants in the propagation of the epidemic according to three scenarii:

- the propagation of the initial virus strain in absence of variants;
- the propagation of the initial virus strain together with its *α* variant;
- the propagation of the initial virus strain together with its *α* and *δ* variants.

**Figure 5:**
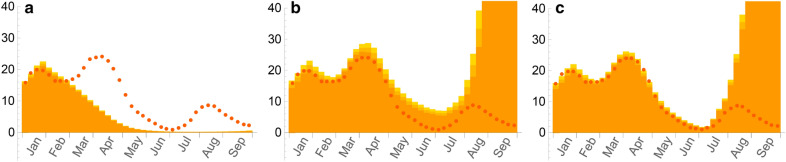
Number of weekly hospital admissions normalized to 50,000 households in three typical scenarii; (**a**) with the SARS-CoV-2 initial strain only and no progressive vaccination campaign; (**b**) with the propagation of the α variant and no progressive vaccination campaign; (**c**) with the propagation of the α and δ variants and progressive vaccination campaign up to the beginning of the δ variant propagation only. The red points correspond to epidemiological data from Santé Publique France^18^ and statistical errors at 95% CL are represented by slightly different orange shades.

Figure 5 shows the number of weekly hospital admissions normalized to 50,000 households for these three scenarii, with three different vaccination strategies. On Fig. 5a, we observe a rapid decrease of the hospital admissions starting from February 2021, even in absence of vaccination. The natural immunity of already infected persons, together with a reduced value of the intensity of social relationships through *NPIs* and favorable temperature effect, is however not enough to completely stop the epidemic. It starts to propagate again, although very slowly, at the end of September 2021 with relaxing social distancing steps. The propagation of the *α* variant is responsible for the third wave in April 2021, as shown on Fig. 5b. In absence of a vaccination campaign, and because of the increased infectiousness of this variant, the epidemic would have propagated exponentially just at the beginning of summer time, when the intensity of social relationships increased significantly, together with the increase of travels over the whole territory. The effect of a progressive vaccination campaign in March, April, May and June is clearly shown on Fig. 5c, as compared to Fig. 5b. This campaign is however still not enough to completely stop the propagation of the *δ* variant since it starts to increase exponentially if the vaccination campaign is stopped together with the increase of travels and social exchanges during summer.

These results clearly show the importance of the social behavior of the population in order to be able to predict the influence of a progressive vaccination campaign. When comparing Fig. 5c with Fig. 2, we can see that a continuous vaccination campaign during summer, together with the natural immunity progressively acquired by the infected persons, are enough to completely stop the propagation of the *δ* variant, eventhough its infectiousness is much higher than that of the *α* variant. Note also that these simulations do not suppose any new *NPIs* in addition to those taken during summer 2020. According to our model, the population fraction either vaccinated or already infected and therefore naturally immunized is around 20% at the beginning of the α variant propagation, and 55% at the beginning of the *δ* variant propagation.

### Influence of the strength of social relationships on the dynamics of the epidemics

**Figure 6:**
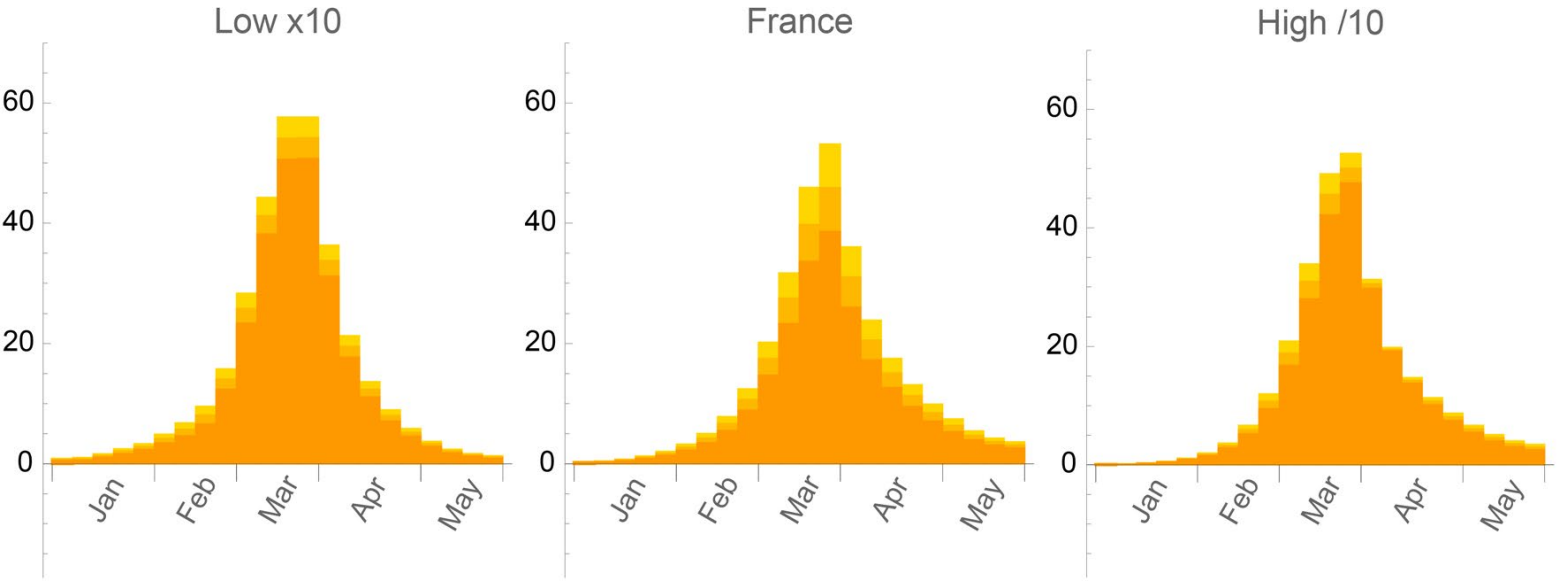
Number of weekly hospital admissions normalized to 50,000 households in France and in two configurations with Low (multiplied by 10), and High (divided by 10) level of social relationships, from left to right respectively, in a typical scenario with no Super Spreading Event, with the same initial conditions and with the same restrictions from the first confinement as in France. Statistical errors at 95% CL are represented by slightly different orange shades.

From the phase diagram indicated on Fig. 1a, we can see that the points representing the social behaviors in France and in the two configurations *Low* and *High* are all in the percolation zone at the beginning of the epidemic, but in rather different positions. In order to illustrate the influence of these different social behaviors on the propagation of the virus, we consider the evolution of the epidemic till May 2020 with the same confinement restrictions as in France. This scenario highlights the essential role played by the density of the social relationships for these configurations, prior to the subsequent changes of these behaviors following the changes in *NPIs* enforced by the authorities. The evolution of the epidemic, as shown on Fig. 6, is a direct consequence of the different position of the points associated to these configurations in the phase diagram of Fig. 1a. This induces a very slow propagation of the epidemic when the strength of the social relationships, both its density and its intensity, is low, and a much faster one when it is high. Although all points associated to these configurations in the phase diagram lie in the percolation zone, the time needed to get an infection propagating on a large scale depends on how far they are from the critical line. Any new enforced *NPI*, like a confinement period for instance, will thus stop the epidemic at a different stage of its propagation, very soon when the strength of social relationship is low and much later when it is high. These patterns correspond qualitatively to what has been observed for instance in Germany and Italy during the first few months of 2020^13,19^.

### Perspectives

The *PERCOVID* model provides a simplified but realistic account of the *COVID*-*19* pandemic in metropolitan France. This model emphasizes the importance of the local social environment of each person in a household. This local environment is well accounted for in a site-bond percolation model in terms of the density of social relationships and their intensity. This leads in particular to a sharp percolation transition which may be used as a guiding principle in order to settle appropriate *NPIs* to reduce, and ultimately stop, the epidemic. Our study should be considered as a very first stage of a new paradigm in order to understand epidemics propagating through respiratory tract or by skin contacts in human populations, complementing the usual modelisation strategies of epidemics.

From the results presented in this work and the first experience gained in the determination of the parameters associated to the social behavior of the French population, we can make the following statements for the expected situation in autumn 2021. Although any new variant may have an increased infectiousness as compared for instance to the *δ* variant, it can only spread on a large scale if there is enough space to propagate around each infected person. This should happen if any of the two following conditions is met:

- the variant escapes largely from the protection of the vaccination and of the natural immunity acquired by the already infected persons;
- there is no continuity of the vaccination strategy, if needed from epidemiological studies, like for instance no third dose to extend the period of immunity for the already vaccinated persons or no vaccination for the already infected persons.

Our model is a first attempt in incorporating explicitly the density and intensity of social relationships for the understanding of epidemic propagating through respiratory tract or by skin contacts in human populations. These parameters have been adjusted to account for the full evolution of *COVID-19* pandemic from December 2019 to Autumn 2021. They should however be extended in forthcoming studies incorporating for instance a stratification in age in each household in order to be able to make accurate predictions on admissions in Intensive Care Units.

## Supplementary Information

Supplementary Information.
