# Supplementary material for: Highlighting the impact of social relationships on the propagation of respiratory viruses using percolation theory
Source: Sci Rep. 2021 Dec 21;11:24326. doi: 10.1038/s41598-021-03812-9 (PMC8692486; doi:10.1038/s41598-021-03812-9)
Supplement: Supplementary file 1 — Supplementary Information. [file 41598_2021_3812_MOESM1_ESM.pdf]

Highlighting the impact of social relationships on the propagation of respiratory viruses using percolation theory

Jean-François Mathiot [a], Laurent Gerbaud [b], Vincent Breton [a]*

a) Laboratoire de Physique de Clermont, Université Clermont Auvergne, CNRS/IN2P3 F-63000 Clermont-Ferrand, France; jean-francois.mathiot@clermont.in2p3.fr

b) Institut Pascal, CHU Clermont-Ferrand, SIGMA Clermont, Université Clermont Auvergne, CNRS F-63000 Clermont-Ferrand, France

Supplementary Information

Parameters of the model

We classify the various parameters of our model into three categories: the epidemiological parameters, the parameters associated to the social behavior of the population in the territory under study and the parameters associated to the mobility of the persons travelling to or within this territory. These parameters are presented in *Supplementary Table S1*. The rate of intra-household infections is determined by the value of q_i and the number N_i of daily intra-household social contacts leading to a potential infection. By construction, we take $q_i = 1$ as intra-household interactions are the closest. This fixes the general scale for the determination of the intensity of social relationships. The percentage of intra-household infections, as compared to the total number of infections computed in our model, was about 23% just before the first confinement period in metropolitan France, in good agreement with the literature [20].

The parameter p associated to the density of social relationships is extracted from a population-based contact survey in various European countries[13,14]. For France, the parameter q associated to the intensity of the social relationships is fixed in order to get a consistent description of the *COVID-19* pandemic for more than 20 months. These consistency constraints are strong given that q should take a value between 0 and 1 (maximum for intra-household social contact). We assume also that q should not be too small (less than 0.20 for instance) in order to keep the possibility to have a very strict limitation of social contacts, comparable to those taken by Chinese authorities in Wuhan at the beginning of the *COVID-19* pandemic. These considerations do fix also the value we should take for the *SARS-CoV2* infectiousness r, since the probability for an infected person to contaminate a susceptible one in a daily social interaction is given by $q.r$. To take into account a reduced virulence of the virus during summer season, we introduced a reduction factor f_T, with a gaussian time dependence [15].

The mobility parameters correspond to the changes in mobility trends apart from the daily social contacts within the first or second circle of social relationships. They are therefore difficult to catch from absolute data for overall travels, except when very strict mobility restrictions are imposed by the authorities both for traveling and for the access to the second circle of social relationships, like for instance during the first confinement period in France. During that period, Apple and Google data show that the reduction of mobility has been very large, in good qualitative agreement with our adjustement (see table S4) [21]. Regarding the vaccination campaign in metropolitan France, we adjust the vaccination rate to the monthly number of persons having full vaccination one week before. Vaccine efficiency, relevant for the ability of a vaccinated person to infect someone else, is set to 95% for the *SARS-CoV-2* initial strain and 90% for its variants.

Epidemiological parameters		
(r,r_α,r_δ)	Infectiousness for SARS-CoV2 initial strain and its two variants α and δ	(0.14, 0.25, 0.38) [22,23]
dr_{as}	Reduction factor of the infectiousness for asymptomatic persons as compared to symptomatic ones	0.5 [21,24,25]
(τ_I, σ_I)	Elapsed time from being infected to becoming contagious, with standard deviation	(3.5, 1.5) [26]
(τ_r, σ_r)	Elapsed time from being infected to the end of the contagiousness period, with standard deviation	(14, 3) [25]
$(\tau_r, \sigma_r)^v$	Elapsed time from being infected to the end of the contagiousness period, with standard deviation, for the α and δ variants	(15,3), (16,3)
(τ_h, σ_h)	Elapsed time between infection and hospital admission, with standard deviation	(15,3) [27]
(p_h,σ_a)	Fraction of infected persons requiring hospital admission, with standard deviation, for both the initial strain and the α and δ variants	(3.5%,1%) [28]
(τ_s,σ_s)	Incubation time, with standard deviation	(5,3) [29,30]
p_s	Rate of symptomatic persons among infected persons	50 % [24]
f_T	Reduction factor of infectiousness in mid-summer	0.5 [15]
σ_T	Standard deviation for the time dependence of infectiousness	45
Parameters related to the social behavior		
p	Density of daily social relationships	0.5
q	Intensity of social relationships	**0.85**
q_i	Intensity of intra-household social relationships	1
p_c	Rate of symptomatic persons staying at home	50 %
N_i	Number of daily intra-household social contacts leading to a potential infection	5
dq_{2c}	Reduction factor of the intensity of social relationships from the 1st to the 2nd circle	0.8
p_{2c}	Probabililty of having social relationships with the 2nd circle	**1**
Mobility parameters		
N_{ext}	Number of daily infections from foreigners visiting metropolitan France	**0**
ρ_{mob}	Percentage of persons having social contacts outside the 1st and 2nd circle	**5 10^{-3}**
N_{mob}	Number of daily contacts outside the 1st and 2nd circle, for each person	**5**

Supplementary Table S1: list of all the parameters used to describe COVID-19 pandemic in France. Parameters impacted by governmental NPIs are shown in bold face in the last column. References used to fix epidemiological parameters are given in the last column also. All time scales are given in days.

Lattice configuration and initial conditions

Supplementary Table S2 provides details on the configuration of our cubic lattice and the model initial conditions. The initially infected persons from the *SARS-CoV2* strain are randomly generated as symptomatic or asymptomatic with probabilites p_s and (1- p_s) respectively. The infection time is also generated randomly within the period $[-(\tau_r-\tau_i),0]$. In order to account for the expected inhomogeneous distribution of the initially infected persons over the metropolitan French territory in December 2019, we divide our cubic lattice in 3x3x3 sub-regions of equal size. Infected persons are distributed randomly in only five of these 27 regions with a distribution ¼(1,0.9,0.8,0.7,0.6). The number of initial infected persons in our simulation is fixed at 14. For smaller values, large fluctuations in the epidemic time development reduce simulation accuracy. The start of the simulation is adjusted to reproduce the observed peak of hospital admissions during the first wave. Simulating the epidemic evolution in metropolitan France at a scale 1/10 from December 2019 to September 2021 requires about 15 minutes computing time on an Intel Core i3 processor at 1.1GHz clock frequency.

Lattice configuration		
	Scale of the simulation associated to the number of households in metropolitan France (approximately 28,500,000)	1/10
	Relative fraction of households with 1, 2 and 3 or more members	(0.36,0.33,0.31) [30]
Initial conditions		
N_0	Number of initially infected persons, all considered as contagious	14
T_0	Simulation starting day	December 1, 2019

Supplementary Table S2: lattice configuration and initial conditions.

As far as the propagation of *SARS-CoV-2* is concerned, and in the absence of a stratification in age that will be considered in a forthcoming study, we assume that a maximum of three persons in each household are susceptible of being infected. This gives a mean number of 1.95 persons per household and corresponds to a population of about 55,500,000 persons susceptible of being infected at the scale of metropolitan France.

The propagation of all *SARS-CoV2* virus, including the initial strain and the α and δ variants, follows the same algorithm. Variant infectiousness has been increased to reflect their higher contagiosity as documented in *Supplementary Table S1*. The fraction of infected persons requiring hospital admission is however taken the same for the initial strain and the α and δ variants in order to get an overall agreement with both the observed patterns for the weekly hospital admissions and the incidence rate. For the initial conditions of the variants propagation, we fix the number of daily contacts in order to get the variant propagating on a large scale, with a similar inhomogeneous distribution over the metropolitan French territory as compared to the SARS-CoV-2 initial strain. These initial infections are associated to foreigners visiting France. Given the higher infectiosity of the variants, the number of these infections for the α and δ variants corresponds approximately to the number of initial infected persons chosen for the *SARS-CoV-2* initial strain. Once the variant propagates significantly in France, additional infections coming from foreigners visiting the country are negligible. The corresponding parameters are indicated in *Supplementary Table S3*. The initial day of the propagation of the variants is estimated from the official database of variants circulating in France [18].

Parameters	***α variant***	***δ variant***
Event start day	February 13, 2021	June 28, 2021
Event final day	March 13, 2021	July 12, 2021
Number of daily contacts	380	1020

Supplementary Table S3: initial conditions for the introduction of the variants in France.

Time Evolution

Each time step represents one day. This elementary time scale is associated to the definition of the number of mean social contacts per day, as encoded in the parameter *p*. At each time step, we proceed through the following actions:

1. Possible infection in the 1st and 2nd circle as well as intra-household, with symptomatic and asymptomatic rates, for each susceptible person on the lattice.
2. Possible infection outside the 1st and 2nd circle, with symptomatic and asymptomatic rates, for each susceptible person on the lattice.
3. Possible infection all over the country, with symptomatic and asymptomatic rates, from infected foreigners visiting France.
4. Change of status for each person in each household on the lattice according to its internal clock.

Phase diagram associated to the δ variant

The phase diagram associated to the percolation transition depends explicitely on the infectiousness of the virus. It is indicated, for the *SARS-CoV-2* initial strain, on *Figure 1* while *Supplementary Figure S1* shows the phase diagram associated to the δ variant, corresponding to a very high infectiousness. As expected, the percolation zone extends to a much larger domain in this case, and necessitates a higher reduction of the intensity of the social relationships, and/or a higher vaccination coverage of the population in order to escape from this percolation zone.

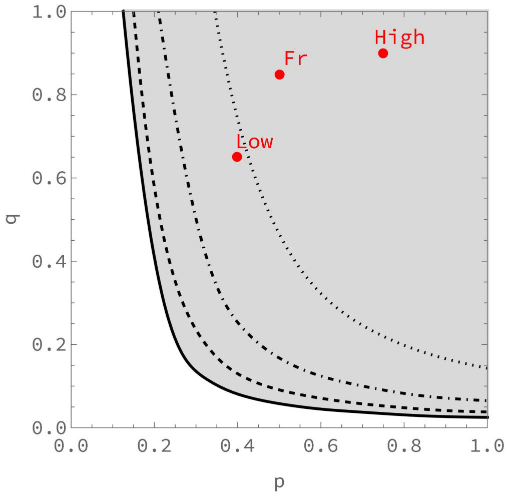

Supplementary Figure S1: phase diagram of PERCOVID for the SARS-CoV-2 δ variant. The full line shows the limit between the non-percolation and percolation zones, in white and grey respectively, in absence of vaccination while the dashed (25% coverage), dot-dashed (50% coverage) and dotted (75% coverage) curves show how the vaccination reduces the percolation zone. The points labeled "Fr", "Low" and "High" correspond to the expected position associated to the social behavior in France and in two configurations with Low and High strengths of social relationships, at the beginning of the epidemic.

Changes of social behaviors in France

We detail in this section how the various changes in the social behavior of the French population, due for instance to the governmental *NPIs*, can be accounted for in *PERCOVID* for the period under study. We do not attempt to get a best fit to the data but rather to have a semi-quantitative understanding of the full evolution of the pandemic in metropolitan France in terms of the changes in the strength of social relationships. We define in these tables $\delta\rho_{mob}$ as the ratio of ρ_{mob} with respect to its value at time $t=0$, as given in *Supplementary Table S1*.

Impact of confinement periods

The confinement periods correspond to strong restrictions in the access to the second circle of social relationships as introduced in our model, and in the mobility and contacts outside the first and second circle. These restrictions were very strict for the first confinement period (March–May 2020), and less strict for the second (November 2020) and third confinements (April–May 2021) in metropolitan France. The corresponding expected changes in $\delta\rho_{\mathrm{mob}}$, N_{mob} and p_{2c} are documented in *Supplementary Table S4*. The indicated time windows correspond to official annoucements [18], as collected for instance in Ref.[32].

Parameters	***1***	***2***	***3***
Event start day	March 17, 2020	October 30, 2020	April 6, 2021
Event final day	May 10, 2020	December 12, 2020	May 2, 2021
q	0.45	0.36	0.25
$\delta\rho_{mob}$	0	0.3	0.5
N_{mob}	0	5	5
p_{2c}	0.1	0.3	0.4

Supplementary Table S4: change of social behaviors and mobility parameters during the three confinement periods.

Epidemic resurgence from the 1st to the 2nd confinement period

The spreading of the *COVID-19* pandemic in France during summer and autumn *2020* is governed by the change of the social behavior of the french population after the first lockdown. This corresponds mainly to an increase of the mobility ouside the first and second circle, as given by the parameters $\delta\rho_{\mathrm{mob}}$ and N_{mob} in *Supplementary Table S5*. The access to the second

circle of the social relationships on the other hand is kept the same, and slightly reduced as compared to *1* in order to account for the restriction in the contacts with elderly. In this study, the time windows corresponding to the change of social behavior should be understood as indicative of a change of social behavior during summer holidays, the start of the academic year, and the boost of the social as well as economic activities in autumn.

Parameters	***1***	***2***	***3***	***4***
Event start day	May 11, 2020	July 12, 2020	August 11, 2020	September 10, 2020
Event final day	July 11, 2020	August 10, 2020	September 9, 2020	October 29, 2020
q	0.25	0.35	0.47	0.56
$\delta\rho_{mob}$	1	20	15	6
N_{mob}	5	10	8	7
p_{2c}	0.9	0.9	0.9	0.9

Supplementary Table S5: change of social behaviors and mobility parameters between the first two confinement periods.

Curfew periods

Between the second and third confinements, the French government enforced curfews. The curfew periods correspond to slight restrictions in the access to the second circle of the (less-essential) social relationships and in the mobility and contacts outside the first and second circle. This translates in slight reductions in $\delta\rho_{\mathrm{mob}}$, N_{mob} and $p_{2\mathrm{c}}$, the rate of these reductions depending on the curfew hour (*8pm* for the first curfew and *6pm* for the second), as indicated in *Supplementary Table S6.* The indicated time windows correspond to official announcements [18], as collected for instance in Ref.[32].

Parameters	***1***	***2***
Event start day	December 13, 2020	January 18, 2021
Event final day	January 17, 2021	April 5, 2021
q	0.31	0.25
$\delta\rho_{mob}$	0.9	0.6
N_{mob}	5	3
p_{2c}	0.7	0.5

Supplementary Table S6: change of social behaviors and mobility parameters during the two curfew periods.

Epidemic evolution since the 3rd confinement (May 2021)

Supplementary Table S7 documents the parameters associated to the progressive change in social distancing after the third confinement period, with a smooth transition to parameters similar to the ones taken for the same summer period in 2020. The parameters in the last column labelled 8 accounts for a relaxation in the intensity of social relationships during autumn 2021.

Parameters	***1***	***2***	***3***	***4***	***5***
Event start day	May 3, 2021	May 19, 2021	June 9, 2021	June 30, 2021	July 21, 2021
Event final day	May 18, 2021	June 8, 2021	June 29, 2021	July 20, 2021	August 10, 2021
q	0.25	0.25	0.25	0.30	0.35
$\delta\rho_{mob}$	0.6	0.7	0.8	10	20
N_{mob}	5	5	5	8	10
p_{2c}	0.5	0.6	0.7	1.0	1.0

Parameters	***6***	***7***	***8***
Event start day	August 11, 2021	September 10, 2021	September 29, 2021
Event final day	September 9, 2021	September 28, 2021	-
q	0.40	0.60	0.75
$\delta\rho_{mob}$	15	6	6
N_{mob}	8	7	7
p_{2c}	1.0	1.0	1.0

Supplementary Table S7: change of social behaviors and mobility parameters after the last confinement period.

Super Spreading Event

A debated issue is whether a *SSE* has played an important role in the emergence of the pandemic in France. The *SSE* which is relevant to our study corresponds to the religious gathering which took place in Mulhouse [17] in February *2020*. In this study, we mimic this event by extra contacts from the participants of the meeting, in a restricted sub-region of the lattice corresponding to the size of the city. The parameters of this event are shown in *Supplementary Table S8*. The initial rate of infected participants, as well as the number of extra daily contacts, are deliberately taken as high in order to get an upper limit for the infection.

Parameters	
Event start day	February 17, 2020
Event final day	February 22, 2020
Initial infectious rate of the meeting participants	10 %
Total number of participants	2,000
Total population of Mulhouse city	110,000
Number of extra daily contacts per participant during the event	100

Supplementary Table S8: parameters describing the SSE which occurred in Mulhouse (France) in February 2020.

In view of the early spread of the *COVID-19* pandemic in France [16,17], the number of infected participants involved in this event is relatively small at the scale of France. In our simulation for instance, we consider that already 140 persons were infected on December 1, 2019. According to our study, this event had therefore no significant impact on the epidemic nation-wide, but a very significant one on the local spread of the epidemic in Mulhouse and in the east of France during that period.

Mean number of secondary contaminations per infected person

Apart from the calculation of the effective reproduction number R, we can also calculate in our model the distribution of the number of secondary infection one infected person can induce during the whole period it is contaminant. The corresponding histogram is shown on *Supplementary Figure S2*.

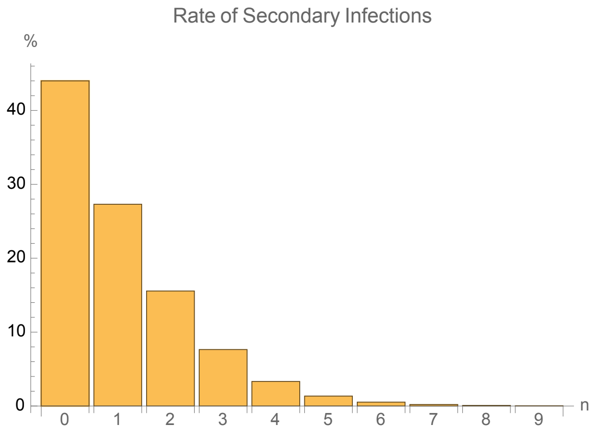

Supplementary Figure S2: distribution of the number of secondary infection per infected person; n corresponds to the number of secondary infections one infected person can induce during its contamination period.

References

20. Madewell, Z.J., Yang, Y., Longini Jr, I.R., Halloran & M.E., Dean, N.E. Household Transmission of SARS-CoV-2, A Systematic Review and Meta-analysis, *JAMA Network Open.* **3(12)**:e2031756; doi.org/10.1001/jamanetworkopen.2020.31756 (2020).

21. Nouvellet, P., Bhatia, S., Cori, A. *et al.* Reduction in mobility and COVID-19 transmission. *Nat Commun* **12,** 1090; 10.1038/s41467-021-21358-2 (2021).

22. Volz, E. *et al.* Assessing transmissibility of SARS-CoV-2 lineage B.1.1.7 in England, *Nature* (2021).

23. Davies, N.G. *et al*. Estimated transmissibility and impact of SARS-CoV-2 lineage B.1.1.7 in England, *Science* **372**, 149 (2021).

24. Oran, DP & Topol, EJ. Prevalence of Asymptomatic SARS-CoV-2 Infection : A Narrative Review, *Ann Intern Med*. **173(5)**, 362-367; doi.org/10.7326/M20-3012 (2020).

25. Byambasuren, O. *et al.* Estimating the extent of asymptomatic COVID-19 and its potential for community transmission: systematic review and meta-analysis, *J Assoc Med Microbiol Infect Disease Canada (JAMMI),*. **4**, 223-234; doi.org/10.3138/jammi-2020-0030 (2020).

26. Byrne, A.W. *et al*. Inferred duration of infectious period of SARS-CoV-2: rapid scoping review and analysis of available evidence for asymptomatic and symptomatic COVID-19 cases, *BMJ Open*. **10(8):**e039856; doi.org/10.1136/bmjopen-2020-039856 (2020).

27. Faes, C. *et al.* Time between Symptom Onset, Hospitalisation and Recovery or Death: Statistical Analysis of Belgian COVID-19 Patients, *Int J Environ Res Public Health*. **17(20)**, 7560; doi.org/10.3390/ijerph17207560 and references therein (2020).

28. Salje, H. *et al.* Estimating the burden of SARS-CoV-2 in France, Science **369**, 208-211 (2020).

29. Lauer, S.A. *et al.* The Incubation Period of Coronavirus Disease 2019 (COVID-19) From Publicly Reported Confirmed Cases: Estimation and Application, *Ann Intern Med*. **172(9**), 577-582 (2020).

30. Yang, L. *et al.* Estimation of incubation period and serial interval of COVID-19: Analysis of 178 cases and 131 transmission chains in Hubei province, China, *Epidemiology and Infection, **148***, E117 (2020).

31. INED, https://www.ined.fr/fr/tout-savoir-population/chiffres/france/couples-menages-familles/menages/

32. Shen, Y., Powell, G., Ganser, I. *et al.* Monitoring non-pharmaceutical public health interventions during the COVID-19 pandemic. *Sci Data* **8,** 225; doi.org/10.1038/s41597-021-01001-x (2021).
